# White matter structural changes in the visual pathway of thyroid-associated ophthalmopathy patients: a free water and multi-shell diffusion imaging study

**DOI:** 10.3389/fneur.2025.1598510

**Published:** 2025-06-27

**Authors:** Jiaqi Yao, Xinjian Lu, Jingxu Ma, Lu Hao, Ying Liu, Xiaopan Huang, Jun Liu, Boding Tong

**Affiliations:** ^1^Department of Radiology, The Second Affiliated Hospital of Xinjiang Medical University, Ürümqi, China; ^2^Department of Radiology, The Second Xiangya Hospital of Central South University, Changsha, China; ^3^Department of Radiology, The Fifth Affiliated Hospital of Xinjiang Medical University, Ürümqi, China; ^4^Department of Radiology, Shaoxing People’s Hospital, Shaoxing, China; ^5^Department of Ophthalmology, The Second Xiangya Hospital of Central South University, Changsha, China

**Keywords:** thyroid-associated ophthalmopathy, free water-diffusion tensor imaging, neurite orientation dispersion and density imaging, tract-based spatial statistics, dMRI (diffusion magnetic resonance imaging)

## Abstract

**Background:**

Compared to single-shell diffusion tensor imaging (DTI), free water (FW) and neurite orientation dispersion and density imaging (NODDI) offer a more comprehensive evaluation of microstructural alterations in cerebral white matter (WM), particularly in detecting crossing fibers. However, research utilizing multi-shell diffusion imaging to investigate thyroid-associated ophthalmopathy (TAO) remains limited. This study employs FW and NODDI to investigate microstructural changes in the white matter of the visual pathways in patients with TAO.

**Methods:**

Multi-shell diffusion magnetic resonance imaging (dMRI) scans were performed on 45 patients with TAO and 31 age- and sex-matched healthy controls (HC). Tract-based spatial statistics (TBSS) analysis was conducted using eight FW and NODDI-derived metrics to identify group differences in white matter microstructure. Furthermore, correlations between these microstructural changes and clinical measures were examined.

**Results:**

TBSS analysis revealed that, compared to HC, patients with TAO exhibited lower free-water corrected fractional anisotropy (fwFA) and free-water corrected axial diffusivity (fwAD), while free-water corrected mean diffusivity (fwMD), free-water corrected radial diffusivity (fwRD), and orientation dispersion index (ODI) were significantly increased (*p* < 0.05, FWE). Notably, ODI demonstrated the highest area under the curve (AUC) among these metrics. Furthermore, fwFA, fwAD, fwMD, fwRD, and ODI showed significant correlations with the Hamilton Anxiety Rating Scale (HAMA), Hamilton Depression Rating Scale (HAMD), and the Graves’ Orbitopathy Quality of Life Questionnaire (GO-QOL2) scores.

**Conclusion:**

This study suggests that abnormalities in the white matter microstructure of TAO patients can be detected through the complementary use of FW and NODDI metrics, and it is revealed that these changes may have an impact on mental health.

## Introduction

Thyroid-associated ophthalmopathy (TAO) is an autoimmune disease characterized by inflammation of the orbital tissues, leading to clinical manifestations such as exophthalmos, diplopia, and visual impairment (1). Notably, 3–5% of patients develop severe complications, including optic neuropathy, which significantly increases the risk of vision loss and suicide (2). The chronic disfigurement and functional impairment associated with TAO impose a substantial psychosocial and economic burden (3), emphasizing the importance of early detection.

Recent evidence suggests that thyroid dysfunction may induce trans-synaptic degeneration in the visual pathways (4). Neuroimaging studies have shown structural changes in both gray and white matter in TAO patients, particularly in regions involved in visual processing (5). For example, diffusion tensor imaging (DTI) has revealed decreased fractional anisotropy (FA) in the lateral geniculate nucleus and optic radiations, indicating microstructural damage to the intracranial visual pathways (6). However, the effect of TAO on the visual cortex—the central hub for visual information integration—remains understudied. This gap is significant, as the bidirectional interaction between the retina and the brain is essential for functional vision, and the integrity of white matter directly influences the efficiency of neural signal transmission (7). Therefore, early monitoring of microstructural changes in white matter related to the visual conduction pathways in patients with thyroid dysfunction is critical for the timely detection of TAO.

Although traditional single-shell DTI is effective in detecting micro structural changes, its accuracy is limited in pathological conditions involving edema or inflammation. These conditions introduce “free water”—extracellular fluid not bound to axons—which can distort diffusion signals and compromise precision. Advanced techniques such as free-water diffusion tensor imaging (FW-DTI) and neurite orientation dispersion and density imaging (NODDI) offer innovative solutions to these challenges (8). FW quantifies extracellular water content (free water fraction, FW) while isolating tissue-specific metrics, such as free-water corrected fractional anisotropy (fwFA), thereby improving sensitivity to axonal integrity (9). NODDI differentiates intracellular, extracellular, and free water compartments, providing more refined micro structural indices such as the neurite density index (NDI) and orientation dispersion index (ODI). While these models have demonstrated significant potential in neurodegenerative diseases, their application in TAO research remains limited. Given its ability to offer detailed biological insights into white matter micro structural alterations, NODDI serves as a highly sensitive and specific tool. Initially, it was employed for the early diagnosis of various brain disorders, including Parkinson’s disease, Alzheimer’s disease, and traumatic brain injury (10–12).

By combining tract-based spatial statistics (TBSS) with multimodal diffusion imaging, this study aims to: (1) characterize white matter microstructural alterations in TAO patients using FW and NODDI metrics; (2) assess the predictive value of these metrics for the early diagnosis of TAO; (3) investigate the correlation between imaging findings and clinical indicators to better understand the underlying pathophysiological mechanisms.

## Methods

### Subjects

This study was approved by the Medical Ethics Committee of the Second Xiangya Hospital of Central South University (Approval No. LYEC2025-0027). All participants voluntarily participated in the study and provided written informed consent. A total of 45 patients with TAO were enrolled in the case group. These patients were diagnosed based on the EUGOGO guidelines (13) and had not received any prior treatment. The inclusion criteria were as follows: (1) Presence of characteristic clinical manifestations, including exophthalmos, eyelid retraction, eyelid edema, diplopia, restricted ocular motility, and optic nerve involvement. (2) Thyroid function status of hyperthyroidism, euthyroidism, or hypothyroidism. (3) Orbital CT or MRI findings consistent with TAO, such as extraocular muscle enlargement (typically sparing the tendons), orbital fat proliferation, or optic nerve compression. Exclusion criteria included: (1) Contraindications to MRI examination. (2) Presence of psychiatric or neurological disorders or other ocular diseases. (3) History of traumatic brain injury, or abnormal intracranial signals detected on routine brain MRI, suggesting underlying intracranial pathology. (4) Illiteracy or significant visual impairment that would prevent completion of neuropsychological assessments. (5) History of alcohol or drug abuse. A control group of 37 healthy individuals, matched for sex, age, and education level, was also included. The inclusion criteria for the control group were: (1) No history of thyroid disease or related ophthalmopathy and no prior ocular surgery. (2) No history of neurological or psychiatric disorders. (3) No abnormal intracranial signals or structural abnormalities detected on brain MRI. (4) No history of alcohol or drug abuse. (5) Ability to successfully complete neuropsychological assessments.

### Clinical assessments

Laboratory assessments were conducted to measure free thyroxine (FT4), free triiodothyronine (FT3), thyroid-stimulating hormone (TSH), and thyrotropin receptor antibody (TRAb). Clinical variables, including age, sex, disease duration, and clinical activity score (CAS), were recorded. For psychological evaluation, the Mini-Mental State Examination (MMSE) (14), the Hamilton Depression Scale (HAMD) (15), the Hamilton Anxiety Scale (HAMA) (16), and the GO-QOL (Graves’ Orbitopathy Quality of Life) scale (17) were administered. The GO-QOL questionnaire is composed of two subscales: one assessing the impact of TAO on visual function, and the other examining the psychosocial consequences of TAO-related appearance changes. It is recognized as the most widely used and validated outcome measure in this field.

### Image acquisition

All participants underwent MRI scanning on a 3.0 T uMR790 scanner equipped with a 32-channel phased-array head coil. Multi-shell diffusion MRI data were acquired using a spin-echo (SE) single-shot echo planar imaging (SS-EPI) sequence with the following parameters: TR/TE = 5,067/71 ms, acquisition matrix = 68 × 68, field of view = 204 × 204 mm^2^, slice thickness = 3 mm, and 45 axial slices. Diffusion weighting was applied with *b*-values of 1,000 and 2,000 s/mm^2^, along 64 diffusion-encoding directions for each *b*-value, in addition to a single *b* = 0 s/mm^2^ (*b*_0_) image. Diffusion images exhibiting severe artifacts, excessive head motion, significant noise, or signal loss were excluded from the analysis.

### Image preprocessing

Diffusion MRI data were preprocessed using the FSL software package on a Linux system through a series of standardized steps. Initially, images with evident artifacts were identified and excluded. Head motion and eddy current distortions were then corrected using the FDT toolbox in FSL, with the *b*_0_ image serving as a reference. This process also included the adjustment of gradient directions to account for eddy current corrections. Next, brain extraction was performed using the BET tool in FSL to remove non-brain tissues and generate a brain mask. DTI parameters, including FA, MD, RD, and AD, were subsequently derived using FSL’s diffusion toolbox. Additionally, the dual-tensor model of DTI was applied to compute FW imaging parameters for all participants, including fwFA, fwMD, fwRD, and fwAD. These calculations were executed using Python scripts on an Ubuntu 20.04 system. Furthermore, NODDI metrics—including NDI, ODI, and FWF—were estimated using the AMICO toolbox.^1^

### Tract-based spatial statistics

Tract-based spatial statistics (TBSS) (18) was performed using the FSL software package. Initially, DTI data were examined to exclude FA images with noticeable distortions or artifacts. Nonlinear registration was then applied to align the FA images to the FMRIB58 FA template. A mean FA skeleton was generated with a threshold of 0.2. Each participant’s FA image was subsequently projected onto this skeleton to construct a white matter skeleton, and all FA images were mapped onto the generated FA skeleton. Additionally, the “tbss_non_FA” command was utilized to apply the same processing steps to FW, fwMD, fwFA, fwAD, fwRD, ODI, NDI, and FWF images. Image visualization was then conducted, maintaining a threshold of 100, and individual parameter values were extracted from the clusters.

### Statistical analysis

Statistical analyses were performed using IBM SPSS Statistics, version 27. Independent samples *t*-tests were conducted to assess continuous demographic and clinical variables, while chi-square tests were applied for categorical variables. The assumption of normality was evaluated using the Shapiro–Wilk test before performing independent samples *t*-tests. A general linear model was established in FSL, incorporating age and gender as covariates. A non-parametric permutation test with a fixed number of 5,000 permutations was employed for variance analysis. Statistical corrections were applied using threshold-free cluster enhancement (TFCE) at the family-wise error (FWE) level, with a significance threshold set at *p* < 0.05. Parameter values from brain regions exhibiting significant differences in the TBSS analysis were correlated with clinical variables and neuropsychological scores. Pearson’s correlation analysis was used when both datasets followed a normal distribution, whereas Spearman’s correlation analysis was applied if at least one dataset did not meet normality assumptions. A threshold of *p* < 0.05 was considered statistically significant. Furthermore, the diagnostic performance of FW and NODDI metrics in TAO patients was evaluated using receiver operating characteristic (ROC) curve analysis, with the area under the curve (AUC) serving as an indicator of discriminatory ability.

## Results

### Study population

A total of 48 TAO patients and 38 healthy controls were initially recruited for this study. Following the screening process, 45 TAO patients and 37 age- and sex-matched healthy controls met the eligibility criteria. Among the TAO patients, exclusions were due to missing clinical information (*n* = 2) and unsuccessful head motion correction (*n* = 1). In the healthy control group, one subject was excluded due to ineffective head motion correction. There were no significant differences between the two groups in gender, age, years of education, or MMSE scores (*p* > 0.05). However, HAMA and HAMD scores were significantly higher in the TAO group compared to the healthy controls (*p* < 0.05) (Table 1).

**Table 1:** Clinicodemographic characteristics of the participants.

	TAO (45)	HC (37)	*Z*/*X*^2^	*p*
Age (years)	43.40 ± 13.30	40.054 ± 9.46	−0.96	0.33^a^
Sex (F/M)	28/17	22/15	0.43	0.51^b^
Disease duration (years)	3.1 ± 2.6	—	—	—
CAS	3.5 ± 1.4	—	—	—
FT4 (pmol/L)	10.0 ± 16.5	—	—	—
FT3 (pmol/L)	5.2 ± 4.5	—	—	—
TSH (mIU/L)	3.0 ± 5.8	—	—	—
TRAb (IU/L)	35.5 ± 92.1	—	—	—
MMSE	29.27 ± 0.11	29.46 ± 0.12	−1.55	0.12^a^
HAMD	8.69 ± 3.97	3.89 ± 2.43	−6.31	<0.001^a^
HAMA	11.60 ± 6.55	3.95 ± 2.25	−6.32	<0.001^a^
GO-QOL (visual functioning)	20.1 ± 3.6	—	—	—
GO-QOL (appearance)	16.2 ± 5.3	—	—	—

CAS, clinical activity score; FT3, free triiodothyronine; FT4, free thyronine; TSH, thyroid-stimulating hormone; TRAb, thyroid-stimulating hormone receptor antibodies; MMSE, Mini-Mental State Examination; HAMA, Hamilton Anxiety Scale; HAMD, Hamilton Depression Scale; QoL, Quality of Life.

a *p*-value with Mann–Whitney test.

b Chi-square test.

### TBSS analysis

**Figure 1:**
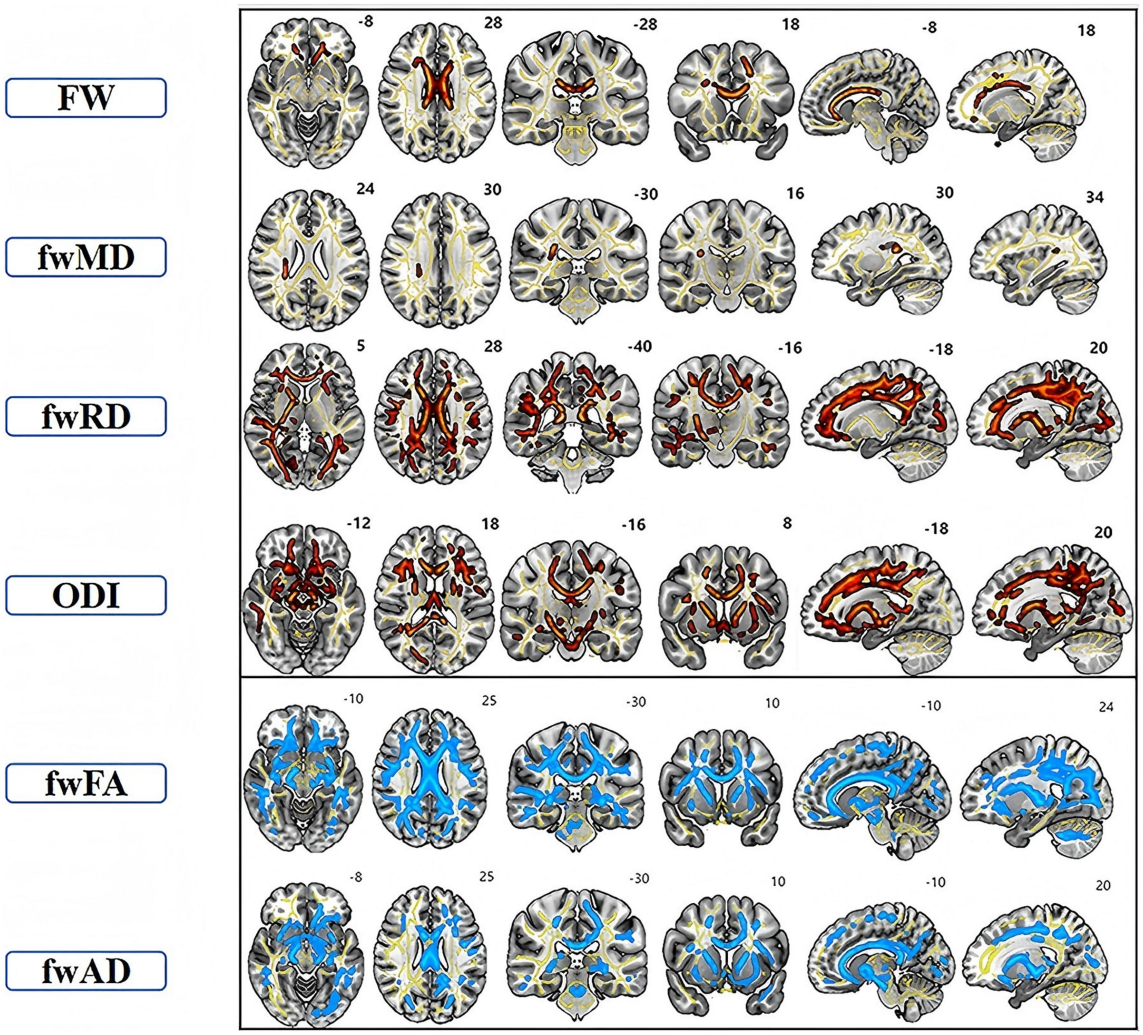
TBSS analysis showed clusters of significant differences in FW-DTI (FW, fwFA, fwMD, fwAD, and fwRD) and NODDI (ODI) metrics between TAO patients and the healthy group (*p* < 0.05, FWE corrected). The superscript number in each image refers to its slice. Red indicates increased indicators in TAO patients compared to HC, while blue signifies decreased indicators. Yellow represents the fiber skeleton.

**Figure 2:**
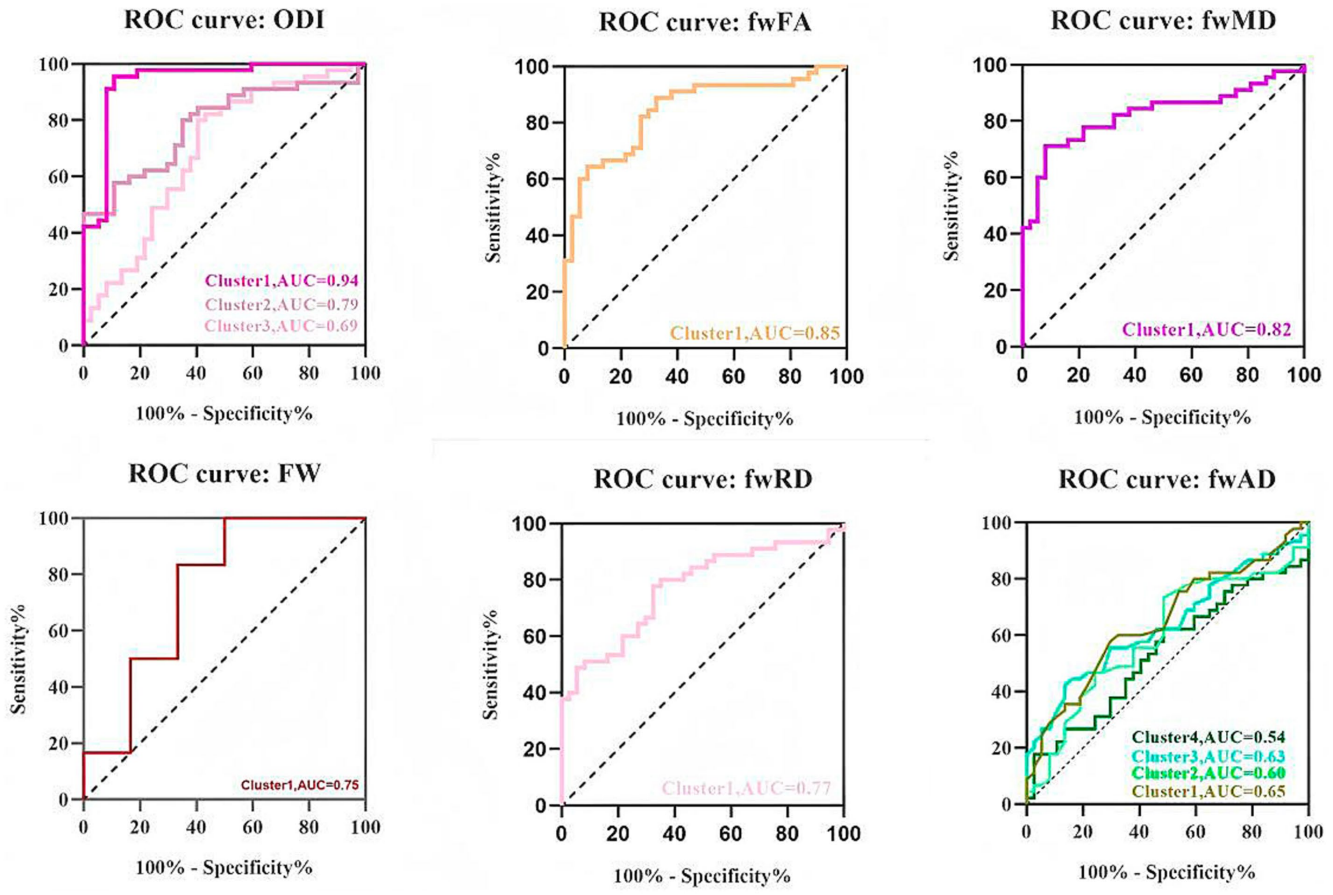
Receiver operating characteristic (ROC) curves of averaged ODI, fwFA, fwMD, FW, fwRD, and fwAD values in each significant cluster identified by tract-based spatial statistics analysis (TBSS). AUC, area under the ROC curve.

The TBSS analysis revealed significant clusters of FW and NODDI metrics. Compared to the HC, the TAO group exhibited significantly decreased fwFA and fwAD, along with markedly increased FW, fwMD, and fwRD. These alterations were predominantly observed bilaterally in the anterior thalamic radiation (ATR), corticospinal tract (CST), cingulum (cingulate gyrus, CGL), splenium and genu of the corpus callosum (F_major and F_minor), inferior fronto-occipital fasciculus (IFOF), inferior longitudinal fasciculus (ILF), superior longitudinal fasciculus (SLF), and uncinate fasciculus (UF). Additionally, ODI values were significantly elevated in the ATR, CGL, F_minor, F_major, IFOF, ILF, and UF regions compared to the healthy controls. Cluster sizes in the TBSS analysis exceeded 10,000 voxels in fwFA (64,300), fwAD (28,566), fwRD (46,715), and ODI (34,819), which were substantially larger than those observed in FA (40,280), AD (2,510), and RD (17,967) (Figure 1 and Table 2). Furthermore, ODI demonstrated the highest statistical significance (*p* < 0.0001). In the ROC curve analysis (Figure 2), the ODI metric yielded the largest area under the curve (AUC = 0.94), with a 95% confidence interval of 0.8822 to 0.9953, a sensitivity of 97.8%, and a specificity of 62.2%.

**Table 2:** White matter tracts in TBSS analysis based on DTI, FW, and NODDI.

Metrics	Voxels	Signal peaks			*p*-value	Brain region
		*X*	*Y*	*Z*		
FA	40,280	9	8	10	0.001	Bilateral: ATR\CST\CGL\F_minor\F_major\IFOF\ILF\UF\SLF
MD	19,093	7	29	7	0.021	Bilateral: ATR\CST\CGL\F_minor\F_major\IFOF\ILF\UF\SLF
RD	17,967	10	21	18	0.015	Bilateral: ATR\CST\CGL\F_minor\F_major\IFOF\ILF\UF\SLF
AD	2,510	35	−15	26	0.002	R: ATR\CST\CGL\IFOF\ILF\UF\SLF
FW	4,671	3	−14	25	0.017	R/L: ATR\CST\CGL\F_minor\IFOF\UF\SLF
fwFA	64,300	−13	39	−16	0.001	Bilateral: ATR\CST\CGL\F_minor\F_major\IFOF\ILF、UF\SLF
fwMD	214	27	−28	22	0.046	R: ATR\CST\IFOF\ILF
fwAD	28,566	−18	−28	52	0.003	Bilateral: ATR\CST\CGL\F_minor\F_major\IFOF\ILF\UF\SLF
	2,285	43	−49	2	0.033	R: ATR\CST\CGL\F_minor\IFOF\ILF\SLF
	1,103	18	9	45	0.023	Bilateral: ATR\CT\CST\CGL\F_minor\IFOF\ILF
	977	35	21	20	0.025	R: ATR\IFOF\SLF\UF
fwRD	46,715	28	−57	14	0.006	Bilateral: ATR\CST\CGL\F_minor\F_major\IFOF\ILF\UF\SLF
ODI	34,819	−28	−3	−23	<0.0001	Bilateral: ATR\CGL\F_minor\F_major\IFOF\ILF\UF
	185	−37	−8	−39	0.043	L: ILF
	138	15	−53	25	0.045	R: ATR\CGL\F_major

R, right; L, left; FA, fractional anisotropy; MD, mean diffusivity; AD, axial diffusivity; RD, radial diffusivity; FW, free-water; fwFA, free-water fractional anisotropy; fwMD, free-water mean diffusivity; fwAD, free-water axial diffusivity; fwRD, free-water radial diffusivity; ODI, Orientation Dispersion Index; ATR, anterior thalamic radiation; CST, corticospinal tract; CGL, cingulum (cingulate gyrus) (hippocampus); F_minor, forceps minor; F_major, forceps major; IFOF, inferior fronto-occipital fasciculus; ILF, inferior longitudinal fasciculus; SLF, superior longitudinal fasciculus; UF, uncinate fasciculus.

### Correlation analysis

**Figure 3:**
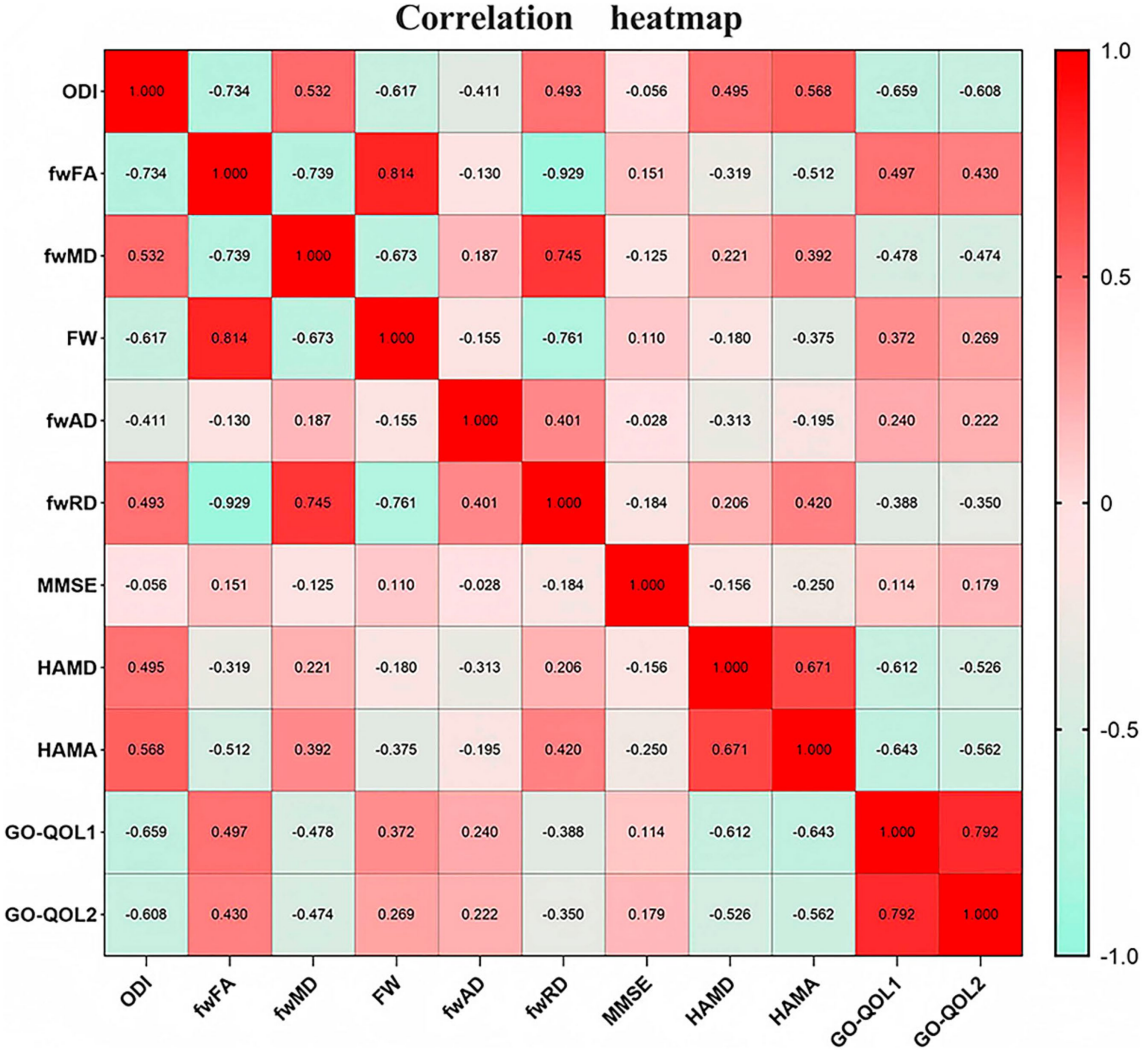
Correlation analysis of indicators with clinical rating scales.

ODI was found to be positively correlated with HAMD (*p* < 0.0001, *r* = 0.495). FW showed a negative correlation with HAMA (*p* < 0.0001, *r* = −0.375) and GO-QOL2 (*p* = 0.03, *r* = −0.319). fwFA was negatively correlated with HAMD (*p* = 0.004, *r* = −0.319), HAMA (*p* < 0.0001, *r* = −0.512), and GO-QOL2 (*p* = 0.042, *r* = −0.304). fwMD was positively correlated with HAMD (*p* = 0.046, *r* = 0.221) and HAMA (*p* = 0.0003, *r* = 0.392). fwAD demonstrated a negative correlation with HAMD (*p* = 0.004, *r* = −0.313). fwRD was negatively correlated with HAMA (*p* < 0.0001, *r* = −0.195) (Figure 3). No significant correlations were observed between any of the indicators and disease duration, CAS score, T3, T4, TSH, or TRAB.

## Discussion

This study represents the first investigation into intracranial white matter microstructural alterations in patients with TAO, employing multishell diffusion imaging techniques. The primary findings revealed significantly reduced fwFA and fwAD, while FW, fwMD, fwRD, and ODI were markedly elevated in various white matter regions in the TAO group compared to the healthy control group. Notably, ODI exhibited the most robust performance. Furthermore, several indicators demonstrated significant correlations with HAMD, HAMA, and GO-QOL2 scores. Following the application of free-water correction, reduced fwFA and fwAD values were observed in patients with TAO across multiple white matter tracts, including the bilateral anterior thalamic radiation (ATR), corticospinal tract (CST), cingulum (cingulate gyrus, CGL), forceps major and minor (F_major and F_minor), inferior fronto-occipital fasciculus (IFOF), inferior longitudinal fasciculus (ILF), superior longitudinal fasciculus (SLF), and uncinate fasciculus (UF). These reductions in fwFA and fwAD may indicate microstructural damage to the white matter itself rather than being solely attributable to an edema effect (19). This finding is consistent with the results of Li et al. (6), who investigated TAO using conventional DTI. Our study further revealed that FW, fwMD, and fwRD levels were significantly elevated in patients with TAO compared to HC. The observed increase in FW can be attributed to heightened extracellular water content, which is typically associated with inflammatory exudation, blood-brain barrier disruption, or alterations in the extracellular matrix (20). Elevated fwMD and fwRD values have been observed as indicators of reduced structural integrity in white matter tissue, suggesting myelin damage. Therefore, the increase in FW may serve as a marker of early inflammation, while the elevations in fwMD and fwRD may reflect chronic structural changes following nerve injury (21).

In addition, in this study, elevated ODIs were predominantly observed in the bilateral ATR, CGL, F_minor, F_major, IFOF, ILF, and UF regions. Inflammatory lesions are often associated with axonal swelling or neuroglial responses, resulting in increased irregularity in nerve fiber orientation. The elevation in ODI may be indicative of neuroadaptive remodeling, suggesting that the brain undergoes adaptive changes in response to visual damage associated with TAO. This adaptation may extend beyond the optic nerve, potentially influencing white matter within the visual pathway (22). Conversely, no significant differences in NDI values were identified in the current study, suggesting that substantial neuronal loss or axonal reduction may not have occurred. Furthermore, this observation implies that, even if some axonal reduction is present, compensatory neuroplasticity might be occurring. Neuroplasticity refers to the brain’s ability to reorganize itself by forming new neural connections, allowing it to adapt to changes and recover from injuries (23) thereby adapting to changes in the optic nerve or visual pathways. Therefore, these metrics are complementary in elucidating potential mechanisms of white matter damage in TAO. Therefore, these metrics are complementary in revealing the potential mechanisms of white matter injury in TAO, suggesting that optic nerve damage may propagate along the visual pathway, leading to white matter fiber degeneration and increased water content, thereby providing new insights into the underlying mechanisms of TAO-related neurological micro structural damage. Furthermore, ODI demonstrated the highest statistical significance and largest AUC in ROC curve analysis, indicating that ODI may be more sensitive than FW metrics. NODDI holds promise as a white matter-specific biomarker for detecting TAO patients, warranting further investigation.

Finally, correlations between specific brain region indicators and clinical scales were examined, revealing that ODI and fwMD exhibited positive correlations with HAMD, while fwFA and fwAD showed negative correlations with HAMD in the ATR, CGL, F_minor, F_major, IFOF, ILF, and UF regions. These regional fiber tracts are primarily involved in visual information processing, emotion regulation, and cognitive functions (24–26). Potential induction of central nervous system white matter microinflammation resulting from immune-inflammatory responses in patients with TAO (27). This leads to white matter degeneration and the accumulation of free water, disrupting fiber organization and signaling (28). Consequently, it affects prefrontal-limbic modulation, impairing both visual and emotional processing. These effects, primarily concentrated in emotion-regulating white matter pathways, may exacerbate depressive symptoms in patients with TAO (29). Second, in the ATR, CST, CGL, F_minor, F_major, IFOF, ILF, UF, and SLF regions, negative correlations with HAMA were exhibited by FW, fwFA, and fwRD, whereas a positive correlation with HAMA was shown by fwMD. These findings suggest that the affected white matter tracts are primarily involved in emotion regulation, motor function, cognition, and self-perception (30). In TAO patients, nerve fibers may be compromised by immunoinflammatory or metabolic issues, impacting the visual-emotional control pathway (31). This may increase sensitivity to changes in appearance, potentially exacerbating anxiety, which is consistent with previous studies (32). A negative correlation with GO-QOL2, a scale assessing mental health, emotional state, and social functioning, was shown by FW and fwFA (3). This correlation indicates that damage and degeneration of white matter bundles may impair emotional network connectivity, cognitive self-image regulation, and motor coordination, impacting daily activities (33). In summary, TAO affects not only ocular health but also mental well-being through changes in white matter microstructure. As a result, TAO should be considered both an ocular and neurological disorder, requiring a multidisciplinary approach to improve patient quality of life.

The limitations of this study are as follows: First, the sample size was relatively small, and future studies should seek to increase the sample size and conduct subgroup analyses to minimize the potential impact of confounding factors. Second, this study focused solely on the microstructure of the visual pathway using diffusion imaging; future research should integrate functional MRI and multimodal approaches to explore how changes in white matter microstructure influence the functional connectivity of brain emotion networks (34). Finally, as a cross-sectional study, it cannot provide insight into the long-term effects; therefore, future studies should incorporate longitudinal follow-up to investigate the neuroplasticity of damaged white matter, allowing for a deeper understanding of the mechanisms underlying white matter damage in TAO. Overall, our findings provide new insights into white matter damage in the visual pathway of TAO patients, highlighting its association with emotional, cognitive, and motor control impairments, and emphasizing the importance of early multidisciplinary diagnosis to improve quality of life.

## Data Availability

The raw data supporting the conclusions of this article will be made available by the authors, without undue reservation.

## Footnotes

^1^ https://github.com/daducci/AMICO
